# Growth, digestive and absorptive capacity and antioxidant status in intestine and hepatopancreas of sub-adult grass carp *Ctenopharyngodonidella* fed graded levels of dietary threonine

**DOI:** 10.1186/s40104-015-0032-1

**Published:** 2015-08-08

**Authors:** Yang Hong, Weidan Jiang, Shengyao Kuang, Kai Hu, Ling Tang, Yang Liu, Jun Jiang, Yongan Zhang, Xiaoqiu Zhou, Lin Feng

**Affiliations:** Animal Nutrition Institute, Sichuan Agricultural University, Chengdu, 611130 Sichuan China; Fish Nutrition and Safety Production University Key Laboratory of Sichuan Province, Sichuan Agricultural University, Chengdu, 611130 Sichuan China; Key Laboratory for Animal Disease-Resistance Nutrition of China Ministry of Education, Sichuan Agricultural University, Chengdu, 611130 Sichuan China; Sichuan Academy of Animal Science, Animal Nutrition Institute, Chengdu, 610066 China; Chinese Academy of Sciences, Institute of Hydrobiology, Wuhan, 430072 China

**Keywords:** Antioxidant status, Grass carp, Intestinal enzyme activity, Threonine

## Abstract

**Background:**

This study was carried out to investigate effects of threonine levels on growth, digestive and absorptive capacity and antioxidant status in intestine and hepatopancreas of sub-adult grass carp (*Ctenopharyngodonidella*).

**Results:**

Weight gain, specific growth rate, feed intake and feed efficiency were significantly improved by dietary threonine (*P* < 0.05). Intestinal activities of trypsin, chymotrypsin, alpha-amylase, lipase, alkaline phosphatase, γ-glutamyl transpeptidase and creatine kinase took the similar trends. Contents of malondialdehyde and protein carbonyl in intestine and hepatopancreas were significantly decreased by dietary optimal threonine supplementation (*P* < 0.05). Anti-superoxide anion capacity, anti-hydroxyl radical capacity, glutathione content and activities of superoxide dismutase, catalase and glutathione-*S*-transferase in intestine and hepatopancreas were enhanced by dietary threonine (*P* < 0.05).

**Conclusions:**

Dietary threonine could improve growth, enhance digestive and absorptive capacity and antioxidant status in intestine and hepatopancreas of sub-adult grass carp. The dietary threonine requirement of sub-adult grass carp (441.9-1,013.4 g) based on weight gain was 11.6 g/kg diet or 41.5 g/kg of dietary protein by quadratic regression analysis.

## Background

Threonine (Thr) is an indispensable amino acid for fish [1]. Dietary threonine deficiency has been shown to cause poor growth and feed conversion in juvenile Japanese flounder (*Paralichthysolivaceus*) [2], as well as low protein deposition in fingerling Indian major carp (*Cirrhinusmrigala*) [3]. It is well known that fish growth is greatly influenced by food digestion and nutrient absorption [4]. To date, there is only one report regarding the effects of dietary threonine on the digestive and absorptive capacity of fish, which showed that diet threonine improved the activities of trypsin, lipase and alpha-amylase in hepatopancreas and intestine of juvenile Jian carp (*Cyprinuscarpio* var. Jian), as well as the activities of intestinal enzymes related to absorption, including alkaline phosphatase (AP), γ-glutamyl transpeptidase (γ-GT) and Na^+^/K^+^-ATPase [5]. However, the digestive and absorptive capacity of fish varies with its feeding habit [6]. Generally, herbivorous fish have a higher digestive capacity in starch than that of omnivorous and carnivorous species [7]. While relative to omnivorous and carnivorous fish species, the herbivorous fish show a poor digestive capacity in protein and fat [6, 8]. Therefore, effects of dietary threonine on digestive and absorptive capacity may be different among fish with different feeding habits. The present study focused on the effects of threonine on digestive and absorptive capacity of herbivorous grass carp (*Ctenopharyngodonidella*).

The function of fish digestive organ is correlated with its development [9]. Threonine has been shown to improve intestinal folds height in juvenile Jian carp [5], as well as anterior intestinal villus height and serosa thickness in juvenile grass carp [10]. On the other hand, the growth and function of the digestive organs are usually correlated with its antioxidant status [11]. Our laboratory studies indicated that the function of digestive organs of juvenile Jian carp was positively related to antioxidant status by methionine hydroxy analogue [12]. However, no studies have been conducted to investigate the relationship between threonine and antioxidant status of tissues and organs in fish. Generally, reactive oxygen species (ROS) are produced during normal aerobic cellular metabolism [13]. When ROS generation rate exceeds that of their removal, oxidative stress occurs which may induce deleterious effects on cells, such as lipid peroxidation and protein oxidation [13]. Huang et al. [14] reported that free transition metal ions, such as iron, copper and manganese, could induce the formation of hydroxyl radicals via the Fenton-Haber Weiss reaction in biological systems. Chelating iron ions could reduce the formation of hydroxyl radicals in stomach of rats [15]. Threonine chelated with iron and copper ions *in vitro* biochemical assays [16, 17] and manganese ions in liver of rats [18]. Thus, threonine might be able to reduce the formation of hydroxyl radicals in living organisms. On the other hand, pig stomach mucins, which were rich in threonine, could scavenge hydroxyl radicals induced by iron ions *in vitro* biochemical assays [19]. It was found that intestinal mucins of common carp (*Cyprinuscarpio* L.) were rich in threonine [20]. Based on these data, threonine might be able to improve the function of fish digestive organs by increasing free radical scavenging ability.

In fish, ROS are scavenged by non-enzymatic antioxidants and antioxidant enzymes [21]. Glutathione (GSH) is an important non-enzymatic antioxidant compound of fish [22]. However, no studies have been conducted to investigate the relationship between threonine and GSH content in tissues and organs of fish. In rats, GSH synthesis takes place mainly in the liver, which needs the participation of ATP [23]. Ross-Inta et al. [24] reported that threonine increased liver ATP level in rats. As with other aerobic organisms, fish developed diverse antioxidant enzymes including superoxide dismutase (SOD), catalase (CAT), glutathione-*S*-transferase (GST), glutathione reductase (GR) and glutathione peroxidase (GPx) [25]. To date, information regarding the effect of threonine on activities of antioxidant enzyme is not available in fish. Sidransky and Rechcigl [26] reported that dietary threonine increased CAT activity in liver and kidney of rats. E2 p45-related factor 2 (Nrf2) regulates a number of antioxidant enzyme genes in bone marrow stromal cells of mice, including SOD, CAT, GST and GR [27]. It was demonstrated that the phosphorylation of Nrf2 at the threonine residue was involved in Nrf2 activation in lung of mice [28]. Nrf2 was found to exist in zebrafish [29]. Based on these observations, threonine may influence the antioxidant defense of fish digestive organs, which warrants investigations.

Grass carp is one of the most important freshwater fish species in the world [30]. Nowadays grass carp is mainly dependent on aquaculture [31]. The threonine requirement of juvenile grass carp was estimated to 13.7 g/kg diet, corresponding to 36.0 g/kg of dietary protein [10]. However, nutrient requirements may vary with the growth stage of fish. Studies showed that the threonine requirement of fingerling India major carp was higher than that of juvenile India major carp [3, 32]. To date, except for juveniles, the threonine requirement for grass carp at other growth stage has not been estimated. Therefore, it is necessary to evaluate the threonine requirement of sub-adult grass carp.

The principal objective of this research was to determine effects of threonine on growth, digestive and absorptive capacity and antioxidant status in intestine and hepatopancreas of sub-adult grass carp. The optimum dietary threonine requirement for the sub-adult grass carp was also evaluated.

## Materials and methods

### Experimental design and diets

The composition of the basal diet is given in Table 1. Fish meal, casein and gelatin were used as intact protein sources. Fish oil and soybean oil were used as dietary lipid sources. According to Abidi and Khan [33], the amino acid profile of whole chicken egg protein was chosen. Crystalline amino acids were used to simulate the amino acid profile with 280 g/kg whole chicken egg protein, except for threonine. L-threonine was added to the basal diet to provide graded concentrations of 3.9 (unsupplemented diet), 6.4, 8.9, 11.4, 13.9, and 16.4 g threonine/kg diet. According to the method of Ahmed et al. [3], diets were made iso-nitrogenous by adjusting crystalline L-glycine. The pH of diets was adjusted to 7.0 with 6.0 N NaOH, as described by Li et al. [34]. According to Shiau and Lo [35], pellets were produced and stored at −20 °C until used. Threonine concentrations in diets analyzed by HPLC were 3.3 (unsupplemented diet), 5.9, 8.4, 10.9, 13.1 and 15.8 g threonine/kg diet, respectively.

**Table 1:** The composition and nutrient content of the basal diet

Ingredients	Content, g/kg	Nutrient content^f^	Content, g/kg
Fish meal	68.0	Crude protein	280.6
Casein	30.0	Crude lipid	46.8
Gelatin	39.9	Available phosphorus	6.0
Crystalline AA mix^a^	146.4	n-3	10.0
Threonine premix^b^	50.0	n-6	10.0
Glycine premix^c^	100.0		
α-starch	280.0		
Corn starch	34.6		
Fish oil	22.8		
Soybean oil	18.9		
Mineral premix^d^	20.0		
Vitamin premix^e^	10.0		
Ca(H_2_PO4)_2_	22.9		
Choline chloride (500 g/kg)	6.0		
Microcrystalline cellulose	150.0		
Ethoxyquin (300 g/kg)	0.5		

^a^Amino acid mix: lysine, 15.99 g; methionine, 8.18 g; tryptophan, 3.27 g; arginine, 11.80 g; histidine, 7.23 g; isoleucine, 11.82 g; leucine, 18.99 g; valine, 14.24 g; phenylalanine, 12.53 g; tyrosine, 10.00 g; glutamate, 32.32 g

^b^Threonine premix: Per kilogram of threonine premix composition from diet 1 to 6 was as follows (g/kg): L-threonine 0 g, 51.02 g, 102.04 g, 153.06 g, 204.08 g, 255.10 g, and corn starch 1, 000.00 g, 948.98 g, 897.96 g, 846.94 g, 795.92 g, 744.90 g, respectively

^c^Glycine premix: Per kilogram of glycine premix composition from diet 1 to 6 was as follows (g/kg): L-glycine 524.58 g, 508.66 g, 492.75 g, 476.83 g, 460.92 g, 445.00 g, and corn starch 475.42 g, 491.34 g; 507.25 g; 523.17 g; 539.08 g; 555.00 g, respectively

^d^Per kg of mineral premix: FeSO_4_•H_2_O (300 g/kg Fe), 25.00 g; CuSO_4_•5H_2_O (250 g/kg Cu), 0.60 g; ZnSO_4_•7H_2_O (345 g/kg Zn), 4.35 g; MnSO_4_•H_2_O (318 g/kg Mn), 2.04 g; KI (50 g/kg I), 1.10 g; NaSeO_3_ (10 g/kg Se), 2.50 g; MgSO_4_•H_2_O (150 g/kg Mg), 230.67 g. All ingredients were diluted with corn starch to 1 kg

^e^Per kg of vitamin premix: retinyl acetate (500, 000 IU/g), 0.80 g; cholecalciferol (500, 000 IU/g), 0.48 g; DL-α tocopherol acetate (500 g/kg), 20.00 g; menadione (230 g/kg), 0.22 g; cyanocobalamin (10 g/kg), 0.10 g; D-biotin (20 g/kg), 5.00 g; folic acid (960 g/kg), 0.52 g; thiamine hydrochloride (980 g/kg), 0.12 g; ascorhyl acetate (930 g/kg), 7.16 g; niacin (990 g/kg), 2.58 g; meso-inositol (990 g/kg), 52.33 g; calcium-D-pantothenate (900 g/kg), 2.78 g; riboflavin (800 g/kg), 0.99 g; pyridoxine hydrochloride (980 g/kg), 0.62 g. All ingredients were diluted with corn starch to 1 kg

^f^Crude protein and crude lipid contents were measured value. Available phosphorus, n-3 and n-6 contents were calculated according to NRC (1993)

### Feeding trial

All experimental protocols were approved by Animal Care Advisory Committee of Sichuan Agricultural University. Sub-adult grass carp were obtained from the Bai-long Lake Fisheries (Sichuan, China). After acclimatized to the experimental condition for 2 weeks, a total of 600 fish with an average weight of 441.9 ± 2.6 g were randomly distributed into 30 cages (1.4 m × 1.4 m × 1.4 m, a gauze disc (diameter, 0.8 m) was placed on the bottom of each cage to collect uneaten feed). Fish were fed to apparent satiation 4 times per day for 8 weeks. According to Cai et al. [36], uneaten feed was removed at 30 min after feeding, air-dried and weighted to measure feed intake. Water temperature, dissolved oxygen and pH were 25 ± °C, 5.0 ± 0.3 mg/L and 7.5 ± 0.3, respectively.

### Sample collection and analysis

Fish in each cage were weighed at the beginning and the end of the feeding trial. After 12 h of fasting, 15 fish from each treatment were anaesthetized in benzocaine bath (50 mg/L), as described by Berdikova Bohne et al. [37] with a minor modification. The intestine, hepatopancreas and muscle of the fish were quickly removed, weighed and stored at −70 °C until analyzed. Intestine, hepatopancreas and muscle samples were homogenized on ice in ten volumes (w/v) of ice-cold physiological saline solution and centrifuged at 6000 *g* for 20 min at 4 °C, and then the supernatant was conserved at −70 °C for determinations of the protein content and enzyme activities.

The protein content was analyzed according to the procedure described by Bradford [38]. Activities of glutamate oxaloacetate transaminase (GOT) and glutamate pyruvate transaminase (GPT) were determined by methods of Bergmeyer and Bernt [39, 40], respectively. Trypsin and chymotrypsin activities were detected according to Hummel [41]. Alpha-amylase and lipase were assayed according to Furne et al. [42]. AP, γ-GT, creatine kinase (CK) and Na^+^/K^+^-ATPase activities were determined by the procedure described by Bessey et al. [43], Rosalki et al. [44], Tanzer and Gilvarg [45] and Weng et al. [46], respectively. Contents of malondialdehyde (MDA) and protein carbonyl (PC) were determined by the procedure described by Zhang et al. [47] and BaltacIoglu et al. [48], respectively. The anti-superoxide anion (ASA) capacity and anti-hydroxyl radical (AHR) capacity were analyzed by using the superoxide anion free radical detection Kit and hydroxyl free radical detection Kit (Nanjing Jiancheng Bioengineer Institute), respectively. GSH contents were determined according to the method of Vardi et al. [49]. GR activity was determined according to Lora et al. [50]. SOD and GPx activities were detected according to Zhang et al. [47]. Activities of CAT and GST were determined according to Aebi [51] and Lushchak et al. [52], respectively.

### Statistical analysis

Results were present as means ± SD. Data were analyzed with one-way analysis of variance (ANOVA). Differences among dietary treatments were determined using the Duncan’s multiple-range test at the level of *P* < 0.05 through SPSS 18.0 for windows. Growth parameters with significant differences were subjected to second-degree polynomial regression analysis. According to Abidi and Khan [33], quadratic regression analysis was used to estimate optimum dietary threonine requirement of sub-adult grass carp.

## Results

### Growth performance

**Fig. 1:**
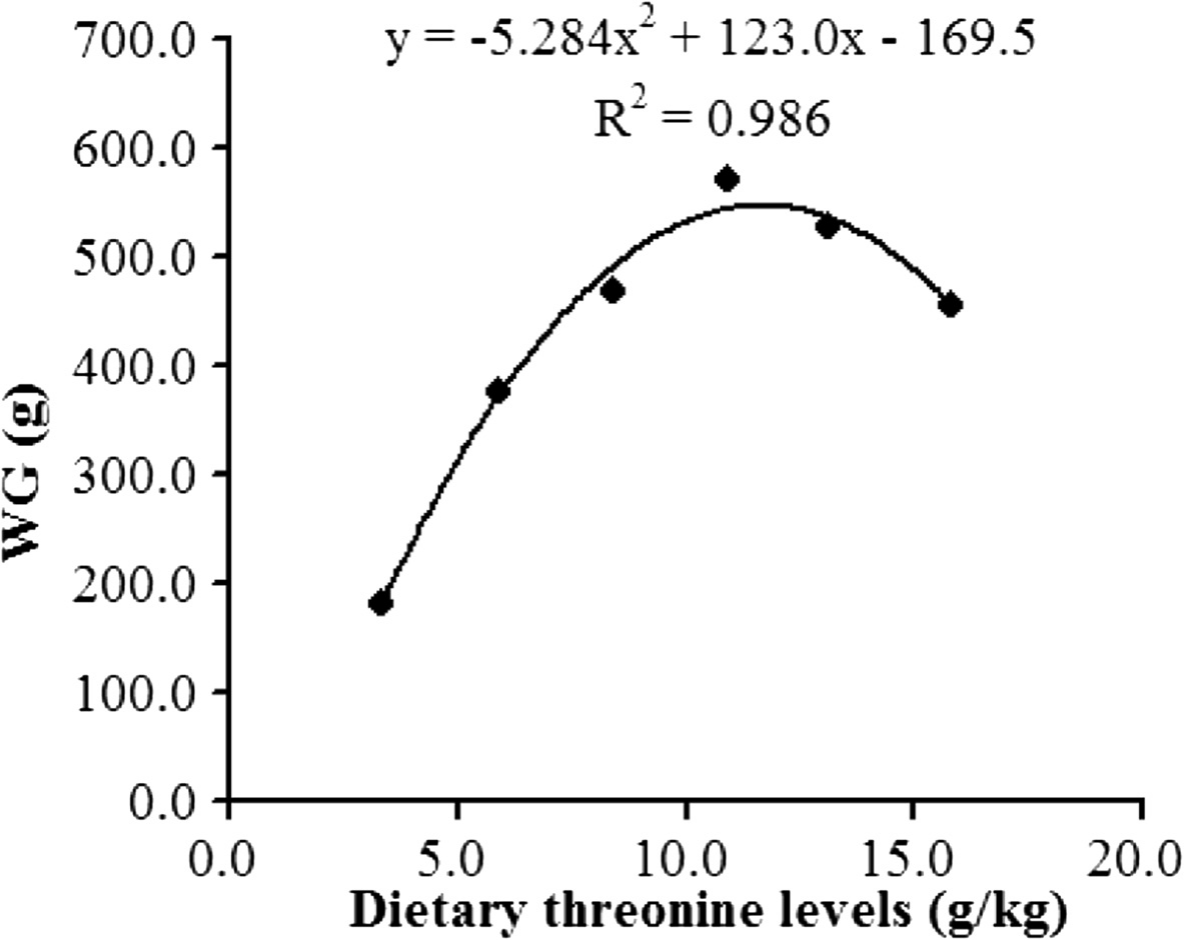
Quadratic regression analysis of weight gain (WG) for sub-adult grass carp (*Ctenopharyngodon idella)* fed diets containing graded levels of threonine for 8 weeks

Effects of graded levels of dietary threonine on growth parameters are given in Table 2, weight gain (WG), specific growth rate (SGR) and feed intake (FI) were significantly improved as dietary threonine levels increased from 3.3 to 10.9 g/kg diet (*P* < 0.05), and decreased thereafter (*P* < 0.05). Fish fed the basal diet (unsupplemented control group) showed the lower feed efficiency (FE) and protein efficiency ratio (PER) compared to those fed threonine-supplemented diets (*P* < 0.05). Regression analysis showed that SGR, FI, FE and PER quadratically responded to increased dietary threonine levels (Y_SGR_ = −0.011X^2^ + 0.277X - 0.160, R^2^ = 0.989, *P* < 0.05; Y_FI_ = −8.729X^2^ + 200.8X - 113.1, R^2^ = 0.989, *P* < 0.05; Y _FE_ = −0.172X^2^ + 4.152X + 28.43, R^2^ = 0.914, *P* < 0.05; Y _PER_ = −0.006X^2^ + 0.148X + 1.013, R^2^ = 0.915, *P* < 0.05). As shown in Figure 1, the dietary threonine requirement of sub-adult grass carp (441.9-1,013.4 g) established by quadratic regression analysis based on WG was 11.6 g/kg diet, corresponding to 41.5 g/kg of dietary protein (Y = −5.284X^2^ + 123.0X - 169.5, R^2^ = 0.986, *P* < 0.05).

**Table 2:** Effects of dietary threonine levels on the growth performance of sub-adult grass carp^g^

Item	Dietary Thr levels, g/kg diet					
	3.3	5.9	8.4	10.9	13.1	15.8
IBW, g/fish	442.0 ± 5.1^a^	441.6 ± 2.3^a^	442.6 ± 1.7^a^	442.2 ± 1.3^a^	441.6 ± 3.0^a^	441.4 ± 2.0^a^
FBW, g/fish	623.4 ± 20.4^a^	817.4 ± 11.1^b^	911.8 ± 26.3^c^	1,013.4 ± 35.2^e^	969.0 ± 28.2^d^	896.6 ± 41.7^c^
WG, g/fish	181.4 ± 19.1^a^	375.8 ± 10.8^b^	469.2 ± 26.4^c^	571.2 ± 34.2^e^	527.4 ± 27.8^d^	455.2 ± 42.8^c^
SGR^h^, %/day	0.61 ± 0.05^a^	1.10 ± 0.02^b^	1.29 ± 0.05^c^	1.48 ± 0.06^d^	1.40 ± 0.05^d^	1.26 ± 0.09^c^
FI, g/fish	464.4 ± 21.3^a^	762.0 ± 14.9^b^	926.1 ± 49.0^d^	1,074.5 ± 29.5^f^	1,023.0 ± 10.6^e^	872.7 ± 5.4^c^
FE^i^, %	38.98 ± 2.44^a^	49.35 ± 2.20^b^	50.83 ± 4.80^b^	53.22 ± 3.94^b^	51.57 ± 2.90^b^	52.17 ± 5.06^b^
PER^j^	1.39 ± 0.09^a^	1.76 ± 0.08^b^	1.81 ± 0.17 ^b^	1.90 ± 0.14^b^	1.84 ± 0.10 ^b^	1.86 ± 0.18 ^b^

IBW: Initial body weight, FBW: Final body weight, WG: Weight gain, SGR: Specific growth rate, FI: Feed intake, FE: Feed efficiency, PER: Protein efficiency ratio

^a,b,c,d,e,f^Means in the same row without a letter in common are significantly different (*P*< 0.05)

^g^Values are mean ± SD (*n* = 5)

^h^Specific growth rate =100 × {[ln (mean final body weight)-ln (mean initial body weight)]/days}

^i^Feed efficiency (%) =100× weight gain (g)/diet intake (g)

^j^Protein efficiency ratio = weight gain (g)/protein intake (g)

### Activities of GOT and GPT in muscle and hepatopancreas

As shown in Table 3, activities of GOT in muscle and hepatopancreas were improved with increasing of dietary threonine levels up to 5.9 g/kg diet (*P* < 0.05). The GPT activity in muscle showed a similar trend with that of muscle GOT activity, and the highest value was obtained when threonine level was 10.9 g/kg diet (*P* < 0.05). However, the GPT activity in hepatopancreas was decreased with increasing of dietary threonine levels up to 8.4 g/kg diet (*P* < 0.05).

**Table 3:** Effects of dietary threonine levels on activities of GOT and GPT in muscle and hepatopancreas of sub-adult grass carp^f^

Item	Dietary Thr levels, g/kg diet					
	3.3	5.9	8.4	10.9	13.1	15.8
Muscle						
GOT, U/g protein	12.34 ± 0.89^b^	13.73 ± 1.14^c^	12.58 ± 1.21^bc^	12.72 ± 1.03^bc^	8.77 ± 0.89^a^	8.24 ± 0.81^a^
GPT, U/g protein	6.26 ± 0.55^a^	11.61 ± 0.53^c^	11.86 ± 0.39^c^	14.41 ± 0.23^e^	13.13 ± 0.47^d^	9.61 ± 0.21^b^
Hepatopancreas						
GOT, U/g protein	27.00 ± 2.32^a^	34.35 ± 2.70^b^	34.68 ± 3.23^b^	35.24 ± 2.10^b^	27.03 ± 2.03^a^	27.09 ± 2.08^a^
GPT, U/g protein	17.38 ± 0.82^c^	16.44 ± 0.89^b^	14.26 ± 0.51^a^	14.78 ± 0.65^a^	14.55 ± 0.62^a^	14.87 ± 1.06^a^

GOT: Glutamate oxaloacetate transaminase; GPT: Glutamate pyruvate transaminase

^a,b,c,d,e^Means in the same row without a letter in common are significantly different (*P*< 0.05)

^f^Values are mean ± SD (n = 6)

### Intestine and hepatopancreas growth

As shown in Table 4, the intestinal length and weight were significantly increased with increasing dietary threonine levels up to 10.9 g/kg diet (*P* < 0.05). Relative gut length (RGL) was not influenced by graded levels of dietary threonine (*P* > 0.05). The intestosomatic index (ISI) of fish fed the basal diet was significantly lower than that of fish fed threonine-supplemented diets (*P* < 0.05). The intestinal protein content (IPC) also followed a similar pattern to that as observed with intestinal length. The hepatopancreatic weight, hepatosomatic index (HSI) and hepatopancreatic protein content (HPC) were significantly improved with the supplementation of dietary threonine (*P* < 0.05), and the maximum values were obtained when threonine levels were 10.9, 8.4 and 8.4 g/kg diet, respectively.

**Table 4:** Effects of dietary threonine levels on IL, RGL, IW, ISI, IPC, HW, HSI, and HPC of sub-adult grass carp

Item	Dietary Thr levels, g/kg diet					
	3.3	5.9	8.4	10.9	13.1	15.8
IL^f^, cm /fish	58.1 ± 5.9^a^	58.5 ± 2.9^a^	61.8 ± 3.2^ab^	63.9 ± 3.5^b^	61.7 ± 3.2^ab^	59.6 ± 5.0^a^
RGL^f^, %	149.0 ± 18.1^a^	144.4 ± 7.6^a^	144.6 ± 8.1^a^	145.5 ± 7.7^a^	145.3 ± 7.7^a^	147.3 ± 10.8^a^
IW^f^, g /fish	6.3 ± 0.8^a^	9.8 ± 1.6^bc^	10.9 ± 1.6^cd^	14.0 ± 2.7^e^	11.8 ± 1.5^d^	9.2 ± 1.0 ^b^
ISI^f^, %	1.04 ± 0.13^a^	1.24 ± 0.21^b^	1.16 ± 0.12^b^	1.26 ± 0.18^b^	1.25 ± 0.15^b^	1.16 ± 0.12^b^
IPC^g^, %	8.33 ± 0.63 ^a^	9.57 ± 1.01^bc^	10.21 ± 1.02^c^	8.90 ± 0.73^ab^	8.71 ± 0.89^ab^	8.49 ± 0.90^ab^
HW^f^, g /fish	12.1 ± 2.1^a^	16.9 ± 2.5^b^	23.7 ± 5.7^c^	26.3 ± 7.1^c^	23.4 ± 3.6^c^	16.5 ± 3.3^b^
HSI^f^, %	1.98 ± 0.27^a^	2.14 ± 0.35^ab^	2.51 ± 0.54^c^	2.37 ± 0.48^bc^	2.49 ± 0.39^c^	2.08 ± 0.36^ab^
HPC^g^, %	9.46 ± 0.50^a^	9.12 ± 0.68^a^	11.61 ± 0.79^c^	10.60 ± 0.65^b^	11.15 ± 0.42^bc^	10.98 ± 0.97^bc^

IL: Intestinal length; RGL: Relative gut length; IW: Intestinal weight; ISI: Intestosomatic index; IPC: Intestinal protein content; HW: Hepatopancreatic weight; HSI: Hepatosomatic index; HPC: Hepatopancreatic protein content

^a,b,c,d,e^Means in the same row without a letter in common are significantly different (*P*< 0.05)

^f^Values are mean ± SD (*n* = 15)

^g^Values are mean ± SD (*n* = 6)

### Activities of intestinal enzymes

As shown in Table 5, intestinal activities of trypsin and alpha-amylase were significantly improved with increasing dietary threonine levels up to 10.9 and 8.4 g/kg diet, respectively (*P* < 0.05), and plateaued thereafter (*P* > 0.05). The highest intestinal activities of chymotrypsin and lipase were obtained when the threonine level was 8.4 g/kg diet.

**Table 5:** Effects of dietary threonine levels on activities of tryspin, chymotrypsin, alpha-amylase and lipase in intestine of sub-adult grass carp^d^

Item	Dietary Thr levels, g/kg diet					
	3.3	5.9	8.4	10.9	13.1	15.8
Trypsin, U/g protein	177.2 ± 10.2^a^	239.9 ± 12.5^b^	252.6 ± 12.1^b^	291.2 ± 16.8^c^	286.1 ± 10.0^c^	294.0 ± 10.6^c^
Chymotrypsin, U/g protein	1.03 ± 0.09^a^	1.00 ± 0.08^a^	2.29 ± 0.15^c^	1.58 ± 0.14^b^	1.45 ± 0.14^b^	1.48 ± 0.12^b^
alpha-amylase, U /mg protein	2.46 ± 0.12^a^	2.51 ± 0.19^a^	2.84 ± 0.09^b^	2.75 ± 0.06^b^	2.73 ± 0.04^b^	2.77 ± 0.04^b^
Lipase, U/g protein	19.11 ± 1.29^ab^	20.97 ± 1.77^bc^	22.71 ± 2.00^c^	22.55 ± 2.41^c^	21.06 ± 2.34^bc^	17.93 ± 1.26^a^

^a,b,c^Means in the same row without a letter in common are significantly different (*P*< 0.05)

^d^Values are mean ± SD (*n* = 6)

As shown in Table 6, activities of AP in proximal intestine (PI), mid intestine (MI) and distal intestine (DI) were significantly improved with the supplementation of dietary threonine (*P* < 0.05), and the highest AP activities were observed for fish fed diets containing 13.1, 8.4 and 5.9 g threonine/kg diet, respectively. Fish fed the basal diet had significantly lower activities of γ-GT in PI and MI compared to those fed threonine-supplemented diets (*P* < 0.05). The highest activity of γ-GT in DI was obtained in fish fed the diet containing 10.9 g threonine/kg diet. Activities of CK in PI, MI and DI were significantly improved with the supplementation of dietary threonine (*P* < 0.05), and the maximum values were obtained when fish fed the diet containing 10.9 g threonine/kg diet. Na^+^/K^+^-ATPase activities in PI and MI were significantly improved with increasing dietary threonine levels up to 5.9 g/kg diet (*P* < 0.05), and gradually decreased thereafter (*P* < 0.05). However, the activity of Na^+^/ K^+^-ATPase in DI was not affected by dietary threonine levels (*P* > 0.05).

**Table 6:** Effects of dietary threonine levels on activities of AP, γ-GT, CK and Na^+^/K^+^-ATPase in intestine of sub-adult grass carp^f^

Item	Dietary Thr levels, g/kg diet					
	3.3	5.9	8.4	10.9	13.1	15.8
AP, mmol of nitrophenol released g/protein per h						
PI	69.5 ± 7.3^a^	84.4 ± 8.1^b^	102.8 ± 10.1^cd^	108.0 ± 10.7^d^	125.0 ± 10.4^e^	92.8 ± 9.1^bc^
MI	110.1 ± 7.0^c^	120.3 ± 13.0^cd^	125.8 ± 4.9^d^	117.6 ± 8.2^cd^	91.9 ± 8.6^b^	71.3 ± 7.4^a^
DI	72.2 ± 6.9^c^	80.3 ± 7.5^d^	69.0 ± 5.4^c^	54.7 ± 5.3^b^	53.8 ± 2.6^b^	35.8 ± 2.8^a^
γ-GT, mmol of 5-amino-2-nitrobenzoate released g/protein per min						
PI	1.82 ± 0.16^a^	2.27 ± 0.15^b^	2.59 ± 0.28^c^	2.52 ± 0.15^c^	3.00 ± 0.16^d^	2.24 ± 0.23^b^
MI	2.12 ± 0.16^a^	2.89 ± 0.26^b^	3.05 ± 0.21^b^	3.09 ± 0.17^b^	2.93 ± 0.19^b^	2.94 ± 0.28^b^
DI	0.73 ± 0.08^a^	0.87 ± 0.11^b^	1.20 ± 0.07^c^	1.23 ± 0.12^c^	1.13 ± 0.11^c^	0.76 ± 0.06^ab^
CK, μmol of phosphorus released g/protein per h						
PI	2.62 ± 0.25^c^	2.72 ± 0.19^c^	2.74 ± 0.24^c^	3.11 ± 0.29^d^	1.52 ± 0.13^b^	1.14 ± 0.13^a^
MI	1.36 ± 0.09^a^	2.40 ± 0.18^c^	2.35 ± 0.17^c^	4.47 ± 0.44^d^	4.28 ± 0.34^d^	1.77 ± 0.17 ^b^
DI	6.69 ± 0.73^a^	7.26 ± 0.44^a^	6.96 ± 0.67^a^	9.35 ± 0.45^b^	8.86 ± 0.70^b^	7.15 ± 0.75^a^
Na^+^/K^+^-ATPase, μmol of phosphorus released g/protein per h						
PI	0.37 ± 0.06^b^	0.42 ± 0.05^c^	0.41 ± 0.02^c^	0.36 ± 0.03^b^	0.37 ± 0.03^b^	0.30 ± 0.03^a^
MI	0.48 ± 0.04^c^	0.62 ± 0.04^d^	0.47 ± 0.03^bc^	0.43 ± 0.04^ab^	0.39 ± 0.03^a^	0.39 ± 0.04^a^
DI	0.35 ± 0.02^a^	0.34 ± 0.02^a^	0.35 ± 0.02^a^	0.36 ± 0.01^a^	0.35 ± 0.03^a^	0.35 ± 0.02^a^

AP: Alkaline phosphatase; γ-GT: γ-Glutamyl transpeptidase; CK: Creatine kinase; PI: Proximal intestine; MI: Mid intestine; DI: Distal intestine

^a,b,c,d,e^Means in the same row without a letter in common are significantly different (*P*< 0.05)

^f^Values are mean ± SD (*n* = 6)

### Antioxidant status in intestine and hepatopancreas

As listed in Table 7, contents of MDA in intestine and hepatoancreas were significantly decreased with increasing threonine levels up to 8.4 and 10.9 g/kg diet (*P* < 0.05), and thereafter increased (*P* < 0.05). The lowest PC contents in intestine and hepatoancreas were observed in fish fed the diet containing 13.1 g threonine/kg diet. ASA capacities in intestine and hepatoancreas were the highest in fish fed diets containing 13.1 and 10.9 g threonine/kg diet, respectively. The AHR capacities in intestine and hepatoancreas were observed in fish fed the diet containing13.1 g threonine/kg diet (*P* < 0.05).

**Table 7:** Effects of dietary threonine levels on MDA, PC, AHR and ASA in intestine and hepatoancreas of sub-adult grass carp^f^

Item	Dietary Thr levels, g/kg diet					
	3.3	5.9	8.4	10.9	13.1	15.8
Intestine						
MDA, nmol/mg protein	2.97 ± 0.23^d^	1.98 ± 0.19^c^	1.33 ± 0.11 ^a^	1.51 ± 0.07 ^b^	1.53 ± 0.11^b^	1.57 ± 0.10^b^
PC, nmol/mg protein	4.37 ± 0.34^c^	3.41 ± 0.25^ab^	3.52 ± 0.23^b^	3.19 ± 0.29^ab^	3.10 ± 0.18^a^	3.34 ± 0.30^ab^
AHR, U/mg protein	120.5 ± 9.9^a^	175.2 ± 12.1^b^	192.9 ± 14.1^c^	266.9 ± 11.1^e^	278.3 ± 12.8^e^	227.6 ± 17.7^d^
ASA, U/g protein	243.5 ± 14.7^b^	253.2 ± 18.3^b^	252.2 ± 17.6^b^	255.9 ± 22.3^b^	278.1 ± 17.2^c^	208.5 ± 14.5^a^
Hepatopancreas						
MDA, nmol/mg protein	2.38 ± 0.19^e^	2.17 ± 0.15^d^	1.57 ± 0.13^b^	1.37 ± 0.13^a^	1.95 ± 0.18^c^	1.92 ± 0.10^c^
PC, nmol/mg protein	6.08 ± 0.41^c^	6.21 ± 0.41^c^	4.99 ± 0.50^b^	5.11 ± 0.36^b^	4.24 ± 0.38^a^	5.90 ± 0.52^c^
AHR, U/mg protein	214.6 ± 14.8^a^	217.2 ± 11.6^a^	218.9 ± 5.4^a^	222.4 ± 7.0^a^	266.1 ± 10.5^b^	224.2 ± 13.3^a^
ASA, U/g protein	220.8 ± 13.8^a^	224.0 ± 19.2^a^	237.2 ± 16.1^a^	264.9 ± 22.8^b^	241.4 ± 22.0^a^	230.4 ± 12.2^a^

MDA: Malondialdehyde content; PC: Protein carbonyl content; AHR: Anti-hydroxyl radical capacity; ASA: Anti-superoxide anion capacity

^a,b,c,d,e^Means in the same row without a letter in common are significantly different (*P*< 0.05)

^f^Values are mean ± SD (*n* = 6)

As listed in Table 8, the supplementation of threonine to certain levels increased GSH contents in intestine and hepatoancreas (*P* < 0.05), the highest GSH contents in intestine and hepatoancreas were observed in fish fed diets containing 13.1 and 10.9 g threonine/kg diet, respectively. Antioxidant enzyme activities in intestine and hepatoancreas were significantly affected by graded levels of dietary threonine (*P* < 0.05). GR activity in the intestine was decreased with increasing the dietary threonine levels up to 8.4 g/kg diet (*P* < 0.05), However, the trend of hepatopancreatic GR activity was opposite to that in intestinal GR. Fish fed the basal diet had a significantly lower activity of intestinal SOD compared to those fed threonine-supplemented diets (*P* < 0.05). The highest activity of SOD in hepatoancreas was found in fish fed the diet containing 5.9 g threonine/kg diet (*P* < 0.05). CAT activities in intestine and hepatoancreas were significantly improved with increasing threonine levels up to 10.9 and 8.4 g/kg diet, respectively (*P* < 0.05), and decreased thereafter (*P* < 0.05). GST activity followed a similar pattern to that as observed with intestinal SOD activity, The highest activity of GST in hepatoancreas was found in fish fed the diet containing 5.9 g threonine/kg diet (*P* < 0.05). The intestinal GPx activity was lower in fish fed the diet containing 15.8 g threonine/kg diet than the other five treatment groups (*P* < 0.05).The GPx activity in hepatoancreas was improved with increasing the threonine levels up to 5.9 g/kg diet (*P* < 0.05), and decreased to a plateau thereafter.

**Table 8:** Effects of dietary threonine levels on GSH content and activities of GR, SOD, CAT, GST and GPx in intestine and hepatoancreas of sub-adult grass carp^e^

Item	Dietary Thr levels, g/kg diet					
	3.3	5.9	8.4	10.9	13.1	15.8
Intestine						
GSH, mg/g protein	1.81 ± 0.16^a^	1.83 ± 0.13^a^	2.56 ± 0.19^b^	3.00 ± 0.19^c^	5.63 ± 0.22^d^	2.98 ± 0.16^c^
GR, U/g protein	23.15 ± 1.22^b^	24.57 ± 1.95^b^	17.05 ± 1.36^a^	19.27 ± 1.78^a^	27.65 ± 2.82^c^	33.59 ± 3.11^d^
SOD, U/mg protein	19.84 ± 1.71^a^	23.87 ± 2.33^b^	23.62 ± 1.13^b^	23.41 ± 2.19^b^	24.83 ± 1.44^b^	23.45 ± 1.84^b^
CAT, U/mg protein	11.70 ± 1.03^a^	16.65 ± 1.48^c^	16.77 ± 1.31^c^	22.59 ± 1.94^d^	18.06 ± 1.26^c^	14.41 ± 1.46^b^
GST, U/mg protein	7.08 ± 0.75^a^	17.41 ± 1.58^b^	16.82 ± 1.48^b^	16.13 ± 1.41^b^	17.36 ± 1.33^b^	17.41 ± 1.58^b^
GPx, U/mg protein	105.4 ± 9.7^b^	109.4 ± 6.6^b^	109.3 ± 4.1^b^	107.4 ± 9.3^b^	104.9 ± 5.7^b^	87.7 ± 8.8^a^
Hepatopancreas						
GSH, mg/g protein	6.13 ± 0.30^b^	7.07 ± 0.41^c^	7.06 ± 0.31^c^	7.83 ± 0.39^d^	6.08 ± 0.29^b^	5.67 ± 0.31^a^
GR, U/g protein	4.08 ± 0.31^a^	13.22 ± 1.02^c^	7.74 ± 0.80^b^	7.91 ± 0.59^b^	7.36 ± 0.73^b^	7.58 ± 0.75^b^
SOD, U/mg protein	83.16 ± 8.65^b^	100.42 ± 6.19^c^	80.39 ± 7.06^b^	84.66 ± 4.00^b^	78.72 ± 5.17^b^	66.57 ± 3.08^a^
CAT, U/mg protein	55.67 ± 5.95^a^	59.11 ± 5.39^a^	79.98 ± 7.11^c^	69.65 ± 7.34^b^	57.94 ± 6.91^a^	56.51 ± 5.61^a^
GST, U/mg protein	22.51 ± 2.05^b^	45.73 ± 2.73^d^	44.27 ± 3.78^d^	25.80 ± 2.68^c^	25.77 ± 2.48^c^	17.34 ± 1.66^a^
GPx, U/mg protein	961.5 ± 79.2^a^	1,205.9 ± 44.5^c^	1,041.0 ± 55.3^b^	1,053.9 ± 57.1^b^	1,026.2 ± 46.4^ab^	995.7 ± 47.2^ab^

GSH: Glutathione; GR: Glutathione reductase; SOD: Superoxide dismutase; CAT: Catalase; GST: Glutathione-*S*-transferase; GPx: Glutathione peroxidase

^a,b,c,d^Means in the same row without a letter in common are significantly different (*P*< 0.05)

^e^Values are mean ± SD (*n* = 6)

## Discussion

In the present study, the growth performance of sub-adult grass carp was significantly influenced by dietary threonine levels. WG, SGR, FI and FE of sub-adult grass carp were significantly improved by dietary threonine, which were in agreement with reports for juvenile grass carp [10], juvenile Jian carp [5] and fingerling Indian major carp [3]. In this study, the improved fish growth may be partly attributed to the promotion of amino acid utilization. It is well known that GOT and GPT play an important role in amino acid metabolism, whose activity can be used to evaluate the utilization of essential amino acids in fish [53]. Results here showed that GOT and GPT activities in muscle, as well as GOT activity in hepatopancreas were significantly increased with optimal threonine supplementation. This was consistent with our previous study [5]. However, GPT activity in hepatopancreas was decreased with the increment levels of dietary threonine up to a certain point. The reason for this result may attribute to the enhanced hepatic gluconeogenesis induced by threonine deficiency. It was reported that threonine deficiency increased hepatic gluconeogenesis in rats [54]. GPT is a rate-limiting enzyme in the conversion of protein to carbohydrate, whose activity in rat liver can be enhanced by the increased hepatic gluconeogenesis [55]. However, this hypothesis needs further investigation in fish. Additionally, the trend of GPT activity in hepatopancreas was opposite with that of our previous study in juvenile Jian carp [5]. The reason for these results is not clear. A possible explanation might be related to the differences in threonine metabolism in different growth stage, as described in terrestrial animals [56, 57]. Further research is needed to clarify this hypothesis. Based on the quadratic regression analysis for WG, the requirement of threonine for sub-adult grass carp (441.9-1,013.4 g) was estimated to be 11.6 g/kg diet, corresponding to 41.5 g/kg of dietary protein.

Fish growth relies on nutrient utilization, which is related to the digestive and absorptive capacity [4]. Generally, fish digestive and absorptive capacity can be reflected by digestive organ growth and development, as well as activities of intestinal enzymes related to digestion and absorption [58]. In our study, there were significant improvements in intestinal length, intestinal weight, ISI and IPC, as well as hepatopancreatic weight, HSI and HPC content. Meanwhile, activities of trypsin, chymotrypsin, alpha-amylase, lipase, AP, γ-GT and CK in whole intestine, as well as Na^+^/K^+^-ATPase in PI and MI were improved by dietary threonine. All these data above suggested that threonine improved the digestive and absorptive capacity of sub-adult grass carp, which were in agreement with our previous study of juvenile Jian carp [5]. It is well known that digestive enzymes in intestinal lumen are mainly secreted from pancreas [59]. Hokin [60] reported that threonine was necessary for the pancreatic alpha-amylase synthesis in pigeons. Meanwhile, threonine increased pancreatic secretion of trypsin, alpha-amylase and chymotrypsinogen in chicks [61]. Besides, threonine was found to be served as an essential component of the active center in γ-GT of rats [62] and CK of chicken [63]. However, the mechanism which threonine improved the digestive and absorptive capacity of fish needs further study.

In fish, the normal function of the digestive organ is correlated with its antioxidant status [11]. The contents of products of lipid peroxidation and protein oxidation, such as MDA and PC, can reflect the antioxidant status of living organisms [64]. In the present study, contents of MDA and PC were decreased with increasing dietary threonine levels up to certain values in both intestine and hepatopancreas, suggesting depressions of the lipid peroxidation and protein oxidation. To date, there were no studies about the effect of threonine on the lipid peroxidation and protein oxidation in fish. Using biochemical *in vitro* assays, it was demonstrated that threonine reduced autoxidation rates of safflower oil in liquid emulsions [65]. As we all know, the lipid peroxidation and protein oxidation are induced by ROS, among which superoxide and hydroxyl radicals are most strongly involved in oxidative damages [13, 66]. In our study, both ASA capacity and AHR capacity in intestine and hepatopancreas were enhanced by dietary threonine, suggesting the improved scavenging abilities against superoxide anion and hydroxyl radicals. To date, information on the relationship between dietary threonine levels and capacity of ASA and AHR has not yet been reported in fish. A possible reason for the improved capacity of AHR might be that threonine enhanced mucin synthesis. Studies showed that intestinal mucin synthesis in piglets [67] and rats [68] were increased by threonine. Meanwhile, *in vitro* biochemical assays, pig stomach mucins could scavenge hydroxyl radicals [19]. Besides, the increased AHR capacity might be also related to the ability of threonine to chelate metal ions. In living organisms, the formation of hydroxyl radicals could be induced by free transition metal ions, such as iron, copper and manganese, via the Fenton-Haber Weiss reaction [14]. In the stomach of rats, the formation of hydroxyl radicals was reduced by chelating iron ions [15]. Threonine was found to chelate with manganese ions in the liver of rats [18] and iron and copper ions *in vitro* biochemical assays [16, 17]. Thus, threonine might be able to decrease lipid peroxidation and protein oxidation in fish digestive organ by improving radical scavenging abilities in these organs, which warrants further study.

In fish, free radicals can be scavenged by non-enzymatic antioxidants, such as vitamin C, vitamin E and GSH [21]. GSH is a direct free radical scavenger in fish [22]. In the present study, both intestinal and hepatopancreatic GSH contents of sub-adult grass carp were increased with optimal threonine supplementation. To date, information on the relationship between dietary threonine levels and GSH contents is limited in fish. Generally, cellular GSH homeostasis is maintained through *de novo* GSH synthesis, glutathione disulfide (GSSG) reduction and uptake of extracellular GSH [69]. In this study, the increased intestinal GSH contents by dietary threonine might be related to the increased uptake of extracellular GSH. It was reported that biliary GSH, which was secreted by liver, was one of the major sources of intestinal GSH in rats [70]. Lauterburg et al. [71] found that an increase in liver GSH content was associated with increased intestinal GSH contents in rats. In terrestrial animals, luminal GSH was uptake by intestine epithelial cells in two ways: (1) be transported intact into cells; (2) be cleaved into glutamate and cysteinylglycine by γ-GT, and then γ-GT transported the cysteinylglycine into the cell for re-synthesis of GSH [72, 73]. In our study, intestinal GSH content was positively related to the γ-GT activity in PI (r = + 0.838, *P* < 0.05), which might suggest that luminal GSH was mainly uptake by intestine epithelial cells of sub-adult grass carp in the second pathway. However, this hypothesis needs further investigation. Liver is the primary site for *de novo* GSH synthesis in rats, which requires the participation of ATP [23]. Ross-Inta et al. [24] reported that dietary threonine increased the liver ATP level of rats. However, whether this ATP synthesis promotion effect of threonine also exists in fish needs study. In the present study, the increased hepatopancreatic GSH content may also be attributed to the promotion of GSSG reduction. GR catalyses the reduction of GSSG back to GSH [74]. Threonine improved GR activity in hepatopancreas of sub-adult grass carp, indicating the improved GSSG reduction. However, the trend of intestinal GR activity was opposite with that in hepatopancreas. A possible reason for this result is that intestinal GR activity was inactivated by GSH. Ogus and Ozer [75] reported that human intestinal GR activity was inactivated by GSH *in vitro*. The reason for GSH not inhibiting GR activity in hepatopancreas might be that GSH in the liver is maintained mainly in the reduced state, and which is highly dependent on GR activity, as it was reported by Kaplowitz et al. [76]. However, further studies are needed to test this hypothesis.

Aside from the antioxidants, antioxidant enzymes, such as SOD, CAT, GST and GPx, also play an important role in protecting cells against free radical damages [13]. The present study showed that threonine enhanced intestinal and hepatopancreatic activities of SOD, CAT and GST, suggesting the improved enzymatic antioxidant ability. To date, few studies have evaluated effects of threonine on activities of antioxidant enzymes in fish. It has been demonstrated that expressions of SOD, CAT and GST are controlled by Nrf2-ARE system in bone marrow stromal cells of mice [27]. Meanwhile, the threonine phosphorylation was involved in Nrf2 activation in lung of mice [28]. Furthermore, the conserved threonine residue was essential for the structure stabilization of Nrf2 in HEK-293 T cells [77]. Kobayashi et al. [29] found that Nrf2 existed in zebrafish. Thus, beneficial effects of threonone on antioxidant enzyme activities might be partly attributed to the enhanced activation of Nrf2. However, this hypothesis needs further investigations. GPx protects cells from excessive levels of H_2_O_2_ and intracellular lipid peroxides by formation of GSSG [78]. In our study, threonine enhanced hepatopancreatic GPx activity of sub-adult grass carp. However, in the intestine, GPx activity was not improved by dietary threonine, but was decreased by excess threonine intake. A possible reason for this phenomenon might be the reduced intestinal mucin synthesis by excess threonine intake. Wang et al. [79] reported that excessive level of dietary threonine reduced mucin synthesis in small intestine of pigs. A decreased content of pig stomach mucins was associated with a decrease of hydroxyl radical scavenging ability *in vitro* biochemical assays [19]. Tabatabaie and Floyd [80] found that GPx of bovine erythrocytes was inactivated by hydroxyl radicals *in vitro*. However, further studies are needed to determine this hypothesis in fish.

## Conclusions

Diets containing the appropriate amount of threonine improved growth, increased digestive and absorptive capacity, and enhanced intestinal and hepatopancreatic antioxidant defense of sub-adult grass carp. Based on the quadratic regression analysis for WG, the requirement of threonine for sub-adult grass carp (441.9-1,013.4 g) was estimated to be 11.6 g/kg diet, corresponding to 41.5 g/kg of dietary protein.

## Abbreviations

AHR: Anti-hydroxyl radical
AP: Alkaline phosphatase
ASA: Anti-superoxide anion
CAT: Catalase
CK: Creatine kinase
DI: Distal intestine
FE: Feed efficiency
FI: Feed intake
GOT: Glutamate oxaloacetate transaminase
GPT: Glutamate pyruvate transaminase
GPx: Glutathione peroxidase
GR: Glutathione reductase
GSH: Glutathione
GSSG: Glutathione disulfide
GST: Glutathione-*S*-transferase
γ -GT: γ -glutamyl transpeptidase
HPC: Hepatopancreatic protein content
HSI: Hepatosomatic index
IPC: Intestinal protein content
ISI: Intestosomatic index
MDA: Malondialdehyde
MI: Mid intestine
Nrf2: E2 p45-related factor 2
PC: Protein carbonyl:
PER: Protein efficiency ratio
PI: Proximal intestine
RGL: Relative gut length
ROS: Reactive oxygen species
SGR: Specific growth rate
SOD: Superoxide dismutase
Thr: Threonine
WG: Weight gain
